# Different Diets Change the Expression of Bovine Serum Extracellular Vesicle-miRNAs

**DOI:** 10.3390/ani9121137

**Published:** 2019-12-13

**Authors:** Suyu Quan, Xuemei Nan, Kun Wang, Linshu Jiang, Junhu Yao, Benhai Xiong

**Affiliations:** 1 State Key Laboratory of Animal Nutrition, Institute of Animal Sciences, Chinese Academy of Agricultural Sciences, Beijing 100193, China; quansuyu@163.com (S.Q.) xuemeinan@126.com (X.N.); cang327@163.com (K.W.); 2 College of Animal Science and Technology, Northwest A&F University, Yanglin 712100, China; 3 Beijing Key Laboratory for Dairy Cow Nutrition, Beijing University of Agriculture, Beijing 102206, China; jls@bac.edu.cn

**Keywords:** feed formula, nonforage fiber source, blood, exosome

## Abstract

**Simple Summary:**

Studies over the last decade have shown that cells can communicate with neighboring or distant cells through complex packets stuffed with selected proteins, lipids, and nucleic acids, called extracellular vesicles. The wrapped macromolecules are miRNAs, which play a central role in mediating the signal communication of creatural patho/physiological systems. Extracellular vesicle-miRNAs vary among species and different body fluids, such as milk, urine, saliva, cerebrospinal fluid and blood, providing general and individual characters of the vesicles. Cow’s milk is significant in the supply of human nutrition. Therefore, the extracellular vesicle-related physiological process of dairy cows should be of concern. This study clarified the miRNA profiling of bovine serum and found their potential influence on immunity. Moreover, we found that different diets could affect miRNA expression. The results implied that people could implement effective dietary strategies to intervene in the physiological state of animals.

**Abstract:**

Cells can communicate with neighboring or distant cells using extracellular vesicles (EVs), mainly attributed to their containing miRNAs. Given that diets can change host circulatory miRNA profiling, and EVs are the major miRNA carriers in serum, we hypothesized that different diets could change bovine circulating EV-miRNA expression. We partly replaced alfalfa hay with whole cotton seed and soybean hull in the feed formula of the tested cows. Blood EVs were isolated using a polyethylene glycol precipitation kit. Particle size analysis revealed exosomes were dominant in bovine serum EVs. Small RNAs were enriched in bovine serum EVs, including miRNAs, snRNAs, tiRNAs, Cis-regulatory elements, piRNAs, etc. In total, 359 types of *Bos taurus* miRNAs were identified by Solexa sequencing. Each cow in the control group contained about 244 types of serum EV-miRNAs, compared to 246 types in the tested group. There were 15 immune-related miRNAs in the top 20 serum EV-miRNAs, accounting for about 80% of the total. Seven differently expressed known miRNAs were detected in responding to different diets. An analysis of the Kyoto Encyclopedia of Genes and Genomes (KEGG) showed differently expressed miRNAs were related to hormone signal pathways and protein metabolism. Bovine serum EVs are abundant with miRNAs, most of which are immune-related. Different diets eventually change the miRNA profiling of bovine serum EVs.

## 1. Introduction

By wrapping high-molecular-weight proteins (e.g., cytosolic, cytoskeletal, plasma membrane and heat shock proteins), lipids (e.g., sphingomyelin, cholesterol, phosphatidylserine and glycosphingolipids), and nucleotides, especially miRNAs, extracellular vesicles (EVs) can mediate intercellular communication in a novel way using these contents [1,2]. EVs are heterogeneous in size (40–2000 nm) and include inward luminal budding of exosomes and outward budding of microvesicles, as well as apoptotic bodies [3]. EVs are not only secreted from hematopoietic origins, particularly by cells of the immune system, including dendritic cells, macrophages, B cells and T cells, but also from non-hematopoietic origins, like neurons and epithelial cells [4]. After being synthesized by these cells, EVs are released into various biological fluids such as blood, saliva, urine, amniotic fluid, and milk, which contribute to many mammalian physiological and pathological processes [5]. Blood bathes all the organs in the body and collects and transports many types of biomolecules secreted, excreted, or discarded by different cells [6]. Serum is an aqueous solution and abundant with dissolved albumin, globulin, enzymes, hormones, nutrients, metabolites and various complex organic molecules, including EVs [5]. Enclosed by a strong phospholipid bilayer membrane, the cargos in serum EVs, especially miRNAs, can deliver at great distances without dilution or degradation. Therefore, various studies have regarded serum EVs as biomarkers with the ability to identify many diseases—such as cancer, immunological disorders, and other systematic disorders in different organs—due to their containing miRNAs [7].

The 18–25 nt non-coding miRNAs can regulate gene expression and control protein synthesis at the post-transcriptional level. After the cleavage of the primary miRNAs (pri-miRNA) and yielding of hairpin precursor miRNAs (pre-miRNA), mature miRNAs capable of performing biological functions are produced [8]. As the key regulatory factors for mammalian growth and development, miRNAs are involved in cell cycling, programmed cell death, cell proliferation, cell differentiation, sensing of nutrient stress and immune processes such as the development of B and T cells, the release of inflammatory mediators, the differentiation of dendritic cells and macrophages, and the proliferation of neutrophils and monocytes [8,9,10]. Specific miRNAs are found in the majority of eukaryotic tissues and organs, and circulatory miRNAs, that are balanced across the entire body, can be free or vesicle-enclosed. Serum EVs are the major miRNA carriers, representing about 78% of total serum miRNAs [11].

The rise of “nutrigenomics” in recent years, which explores the metabolism of nutrients using high-throughput technologies, elucidated another important role of diets besides providing nutrients, namely the regulation of gene expression [12]. It has been proven that changes in nutrient intake induced by different diets such as fatty acids, carbohydrates, amino acids, vitamins, and phytochemicals could regulate the expression of host miRNAs, leading to the modification of host gene expression. Additionally, changing the expression of miRNAs through dietary change has been used as a therapeutic agent to treat some human diseases [13]. Given that diets can change the expression of host circulating miRNAs, and that EVs are the major miRNA carriers in serum, we hypothesized that different diets could change bovine circulatory EV-miRNA expression and, accordingly, designed different feed formula. We partly replaced the alfalfa hay in the bovine feed formula with whole cotton seed and soybean hull to reduce the cost of forage due to high-quality alfalfa being in short supply and costly [14]. On the other side, as high-fiber byproduct feeds, whole cotton seed and soybean hull are recycled forage, conforming to the concept of the sustainable development of agriculture. This study shows that bovine serum EVs were abundant with immune-related miRNAs, and that different diets did change the serum EV-miRNA profiling. It is a great possibility that people could implement effective dietary strategies to intervene in the physiological state of animals.

## 2. Materials and Methods

### 2.1. Ethics Statement

The Holstein cows involved in this experiment were supplied by the Beijing Dairy Cow Center. The study was approved by the State Key Laboratory of Animal Nutrition, the Institute of Animal Science, and the Chinese Academy of Agriculture Sciences, Beijing, China. All operations were performed strictly according to the Directions for the Caring of Experimental Animals from the Ministry of Science and Technology, China (no. 398, 2006). The experimental period was 30 days and the cows had access to water and feed ad libitum throughout the experiment.

### 2.2. Animals and Management 

Six Holstein cows (n = 6101 ± 10 DIM, 574 ± 36 kg BW, 32 ± 2 kg milk/d) were utilized in the experimental period, while housed in 6 environmentally controlled chambers and exposed to a constant temperature of 20 °C, with 40% humidity. The control cows were fed with Total Mixed Ration (TMR), while the tested cows were fed with feed that partly replaced alfalfa hay with whole cotton seed and soybean hull. The TMR formula (Table 1) for the two groups was designed to exceed the predicted requirements (NRC, 2001).

### 2.3. Serum Collection

The blood samples from each cow were collected at 07:00 h on the 30th day of the experimental period. A total of 10 mL of whole blood was collected using a blood collection tube, which should not contain anticoagulants such as EDTA or citrate. The serum was isolated at 3000× *g* for 10 min at room temperature. The serum samples were preserved at liquid nitrogen.

### 2.4. Preparation of EVs from Serum 

The exoEasy Maxi Kit (catalog no. 76064, QIAGEN, Germany) was used for the purification of exosomes and other EVs from cattle serum, depending on a membrane-based affinity binding step. According to the instructions, 4 mL serum was taken as a response system. An amount of 4 mL buffer XBP was mixed with 4 mL sample and warmed to room temperature. The mixture was added to the exoEasy spin column and centrifuged at 500× *g* for 1 min and the flow-through was discarded. An amount of 10 mL buffer XWP was added to the column and centrifuged at 5000× *g* for 5 min and the flow-through was discarded. An amount of 400 μL Buffer XE was added to the membrane and incubated for 1 min. The liquid was centrifuged at 500× *g* for 5 min to collect the eluate, and then the centrifuging was repeated at 5000× *g* one more time.

### 2.5. Nanoparticle Tracking Analysis

Serum pools from the control and tested groups were chosen to carry out this test. Serum mixtures of 3 cows in the control group were pooled together and fed and treated equally. Similarly, 3 cows from the tested group were pooled together. The isolated EVs were analyzed using nanoparticle tracking analysis to identify their physical characterization. The operations were carried out in strict accordance with the operating procedures. The particle size was tested using a sCMOS Nanosight camera with a 405 nm laser (catalog no. NS300, Malvern, UK).

### 2.6. Transmission Electron Microscopy

Serum pools from the control and tested groups were chosen to carry out the test as before. The exosomes were resuspended with 2% paraformaldehyde (catalog no. 158127, Sigma, US) and 5 μL was dropped onto the copper wire and rested at room temperature for 20 min. After being rinsed with 1× phosphate buffered solution 3 times, it was fixed with 1% glutaraldehyde solution (catalog no. G6257, Sigma, US) for 5 min. Then, it was rinsed 10 times with distilled water and negative dye with 5.4% uranyl acetate (catalog no. 22400, Electron microscopy sciences, US) for 5 min. After drying, it was observed using a Transmission Electron Microscope (TEM) (catalog no. FEI Tecnai G2 F20 S-TWIN, US).

### 2.7. Small RNA Library Construction and Sequencing

The serum EV-miRNAs were isolated using the miRNeasy Serum/Plasma Kit (catalog no. 217184, QIAGEN, Germany) following the manufacturer’s instructions. The quality of small RNA was tested by 2% agarose gel electrophoresis and Agilent Bioanalyzer 2100 (catalog no. G2939A, Agilent, CA, US). TruSeq Small RNA Sample Prep Kits (catalog no. RS-200-0012, Illumina, US), Qubit dsDNA Assay Kit (catalog no. Q328520, Life Technologies, US), Qubit RNA Assay Kit (catalog no. Q32852, Life Technologies, US), SuperScript II Reverse Transcriptase (catalog no.18064-014, Invitrogen, US) and DNA loading dye (catalog no.LC6678, Invitrogen, US) were utilized in these steps. Briefly, 3’ and 5’ adapters were ligated to the end of the RNA molecules. Then, the libraries were reverse transcribed, and the polymerase chain reaction was subsequently amplified. The cDNA constructs were purified and recovered, and the libraries were checked and eventually normalized.

### 2.8. Bioinformatics Analysis

The basic reads were converted into raw data/reads by base calling. Low-quality reads, 5’ primer contaminants and poly (A) were removed, and the sequences between 15 and 41 nt were retained. FASTX Toolkit (version 0.0.13) (http://hannonlab.cshl.edu/fastx_toolkit/) and NGS QC Toolkit (version 2.3.2) (https://omictools.com/ngs-qc-toolkit-tool) were used to control the quality of Q20 and filter the reads with N bases. The RNAs were subjected to the GenBank databases (http://www.ncbi.nlm.nih.gov/genbank/) to match the reads with the reference genome (*Bos taurus*, RefSeq assembly: GCF_002263795.1). BLAST (https://blast.ncbi.nlm.nih.gov/Blast.cgi) and Rfam (version 10.1) (http://www.sanger.ac.uk/software/Rfam) were used to remove transcripts, repeat sequences and annotate the non-coding RNAs as tiRNAs, rRNAs, small nuclear RNAs, small nucleolar RNAs, Cis-reg, other Rfam RNAs, etc. The known miRNAs and their structures were identified using miRBase (version 22.0) database (http://www.mirbase.org/). The unannotated sequences were made use of definition new miRNAs with miRDeep2 (https://www.mdc-berlin.de/content/mirdeep2-documentation) and the hairpin-structures were predicted using RNAfold (http://rna.tbi.univie.ac.at/cgi-bin/RNAWebSuite/RNAfold.cgi). The target genes of differentially expressed miRNAs were predicted by miRanda software (http://miranda.org.uk). Gene ontology (GO) (http://geneontology.org) and Kyoto Encyclopedia of Genes and Genomes (KEGG) pathway analyses (https://www.kegg.jp) were performed with the predicted target genes. GO function enrichment took all gene transcriptions/lists as a background and the different miRNA target genes list as a filter of the candidate list. Hypergeometric tests and the Benjamini and Hochberg multiple inspection were utilized to calculate the representative *p* value and their correction. The pathway analysis was conducted using the KEGG database (combined with KEGG annotation results, https://www.kegg.jp), and the significance of enrichments in the pathways was calculated using a hypergeometric distribution test.

## 3. Results

### 3.1. Identification of Bovine Serum EVs

**Figure 1:**
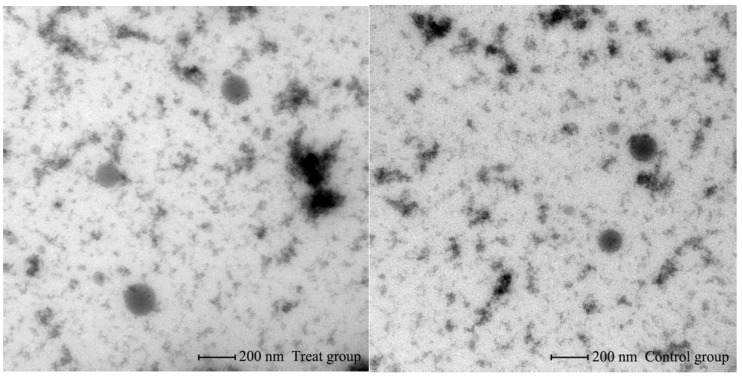
Identification of serum-derived extracellular vesicles (EVs) in Transmission Electron Microscope (TEM).

**Figure 2:**
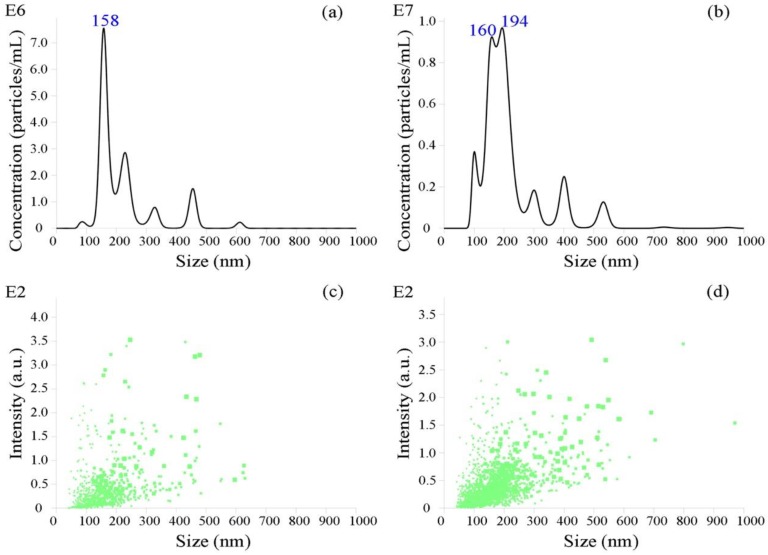
Particle size distribution. (**a**) The concentration of extracellular vesicle (EV)-particles in the control group. (**b**) The concentration of EV-particles in the tested group. (**c**) The intensity of EV-particles in the control group. (**d**) The intensity of EV-particles in the tested group. a.u. means the intensity of the light signal: Arbitrary Unit.

The morphology of the EVs and their particle size distribution were characterized using a Transmission Electron Microscope (TEM) (Figure 1) and Nanoparticle Tracking Analysis (NTA) (Figure 2), respectively. The EVs were shown to be rounded, empty and cup-shaped (ranging from 100 to 200 nm) by the TEM and did not show significant difference between the two groups. The NTA showed that diameter was peaked in 158 nm in the control group and 194 nm in the tested group (Figure 2a,b), respectively. The particle diameter range of the control group was 70–500 nm, while the tested group was 80–560 nm (Figure 2c,d). A large number of particles were distributed around 160 nm in both groups. Less particles were distributed at 200 nm in the control group, while the diameter of tested group peaked at this point. 

### 3.2. Small RNAs Are Abundant in Bovine Serum EVs

The RNA concentration and miRNA percentage are shown in Table 2. The RNA concentrations ranged from 301.1 to 3045.6 pg/uL, while the mean miRNA proportion was 71% in the control group and 62% in the tested group. The results showed that there were small RNAs, especially miRNAs, encapsulated by the EVs in the bovine serum samples.

**Figure 3:**
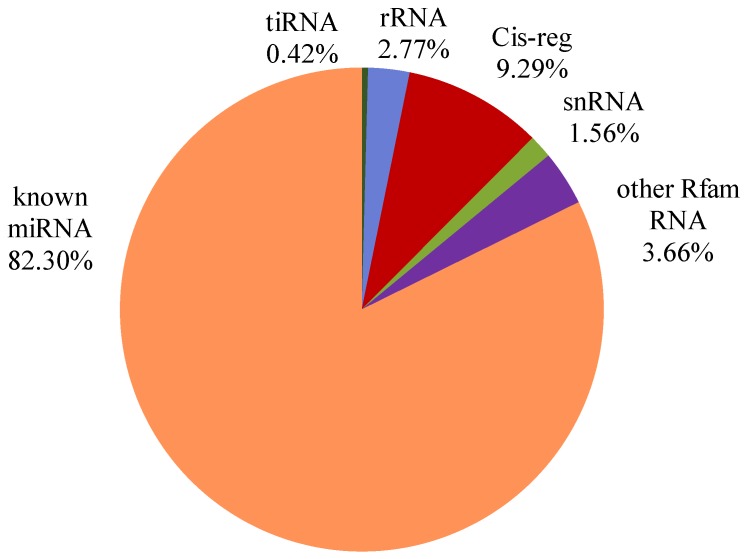
The proportion of annotated small RNA in serum EVs.

The means of raw reads were 27,169,567 in the control group and 30,539,294 in the tested group. The clean reads of all samples were counted between 11.05 and 24.19 M. The genome alignment rate was distributed from 69.20% to 77.06%. Alignment using miRBase (version 22.0) identified a total of 1,537,256 reads, representing 1295 different types of miRNAs identified among all species, with 359 types of known *Bos taurus* miRNAs. The proportion of annotated small RNAs in the serum EVs of all samples is shown in Figure 3. There were many types of small RNAs, including miRNAs, tiRNAs, Cis-regulatory elements (Cis-reg), piRNAs, snRNAs, etc. The annotated known miRNAs accounted for about 80% of the small RNAs, while Cis-reg occupied about 10%. The other Rfam RNAs (such as piRNAs and ribozyme), tiRNAs, snRNAs and rRNAs, made up about 10% in total. The number of known miRNAs of each sample annotated by the *Bos taurus* database was listed in Table 3. The mean kind numbers of miRNAs in the control group and tested group were 244 and 246, respectively. The known miRNAs length distribution of each sample was drawn into a line chart (Figure S1). Most identified miRNAs were distributed between 19–23 nt and the main length of miRNAs in all samples was 22 nt.

### 3.3. Immune-Related miRNAs Are Highly Expressed in Bovine Serum EVs

**Figure 4:**
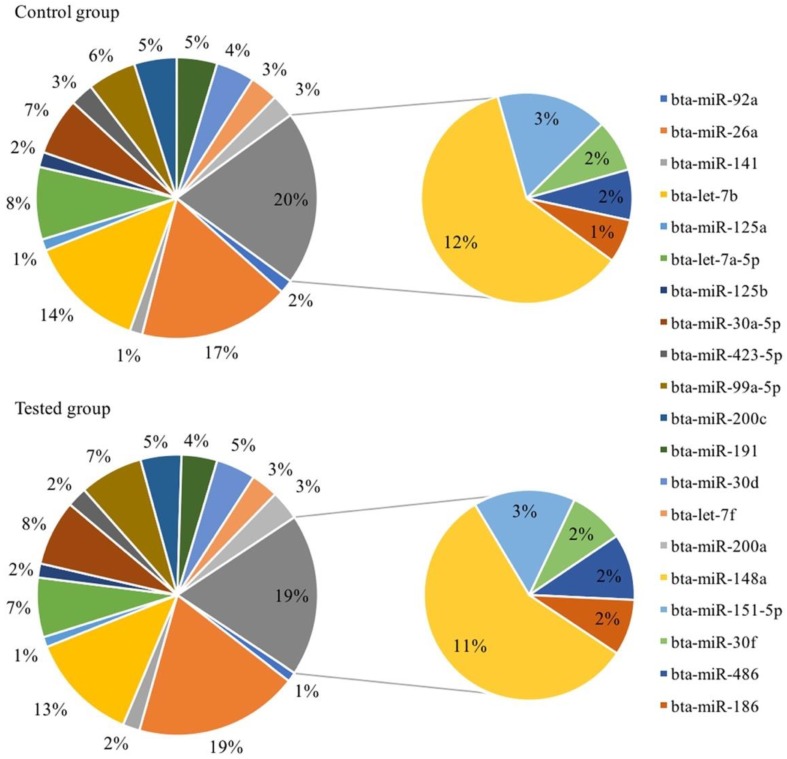
The top 20 highly abundant miRNAs of all samples. The miRNAs in the bigger circle are immune-related, and immune-independent miRNAs are in the smaller circle.

The top 20 miRNAs with the highest expression in the two groups are shown in Figure 4. Fifteen miRNAs from the top 20, accounting for about 80% of the totality, are immune-related, namely bta-miR-26a, bta-let-7b, bta-let-7a-5p, bta-miR-30a-5p, bta-miR-99a-5p, bta-miR-200c, bta-miR-30d, bta-miR-191, bta-let-7f, bta-miR-200a, bta-miR-423-5p, bta-miR-125b, bta-miR-141, bta-miR-92a and bta-miR-125a. In the control group, bta-let-7a-5p ranked fourth, but sixth in the tested group, while bta-miR-30a-5p and bta-miR-99a-5p ranked ahead of it. The 15th bta-miR-30f of the control group decreased to the 18th position in the tested group, while the original 18th bta-miR-141 increased to the 14th position in the tested group. Although there were slight ranking changes in the expression of all miRNAs, the top 20 types of miRNAs were consistent between the two groups, and there were no significant differences in their expression.

### 3.4. Effects of Different Diets on the Expression of EV-miRNAs

**Figure 5:**
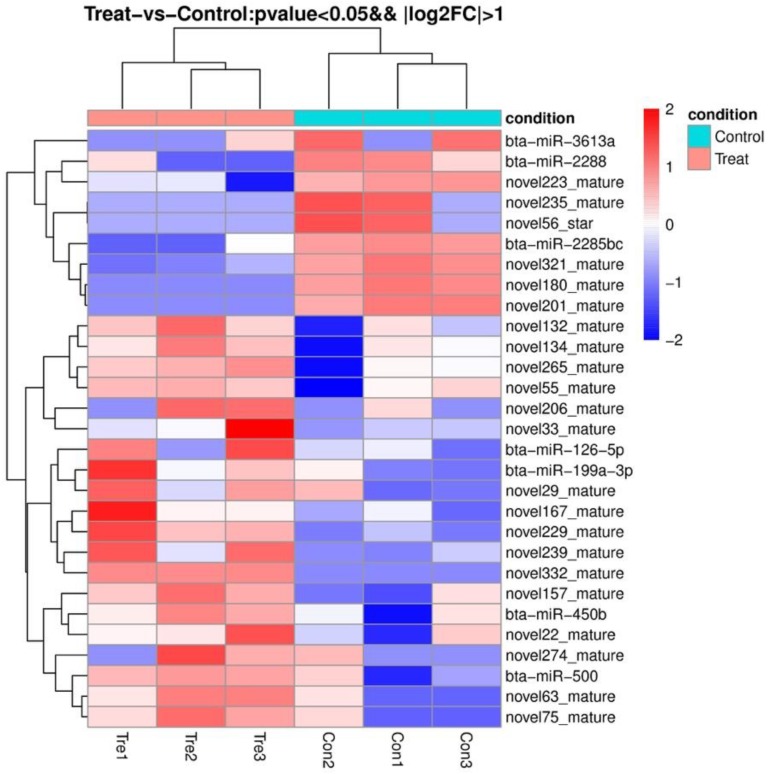
Heat map of differently expressed miRNAs annotated with *Bos taurus* between two groups.

Cluster analysis was performed and the differently expressed miRNAs between the two groups were shown using a heat map (Figure 5). Twenty-two novel miRNAs were differently expressed between the two groups. There were seven differently expressed known miRNAs between the two groups annotated by the *Bos taurus* miRNA database: bta-miR-126-5p, bta-miR-199a-3p, bta-miR-2285bc, bta-miR-2288, bta-miR-3613a, bta-miR-450b, and bta-miR-500 (Table 4). Among them, bta-miR-2285bc, bta-miR-2288 and bta-miR-3613a were specific in the control group, while bta-miR-450b was specific in the tested group.

**Figure 6:**
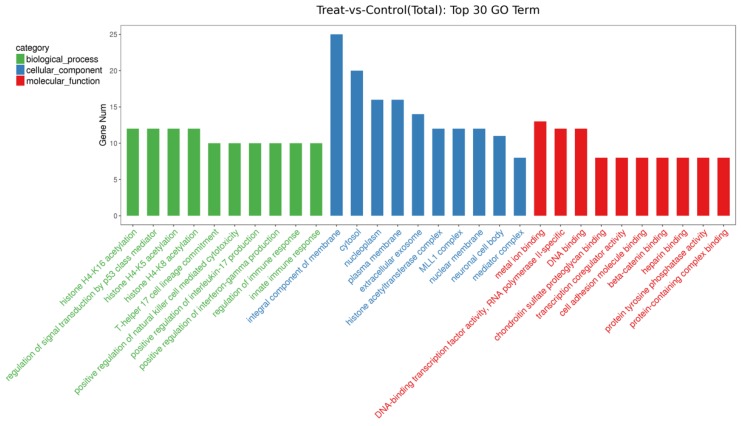
Gene ontology (GO) enrichment of the top 10 target genes of differently expressed known miRNA in three categories. The target genes of differently expressed known miRNAs were classified in terms of biological process (the green columns), cellular components (the blue columns), and molecular function (the red columns), arranged from large to small, respectively.

**Figure 7:**
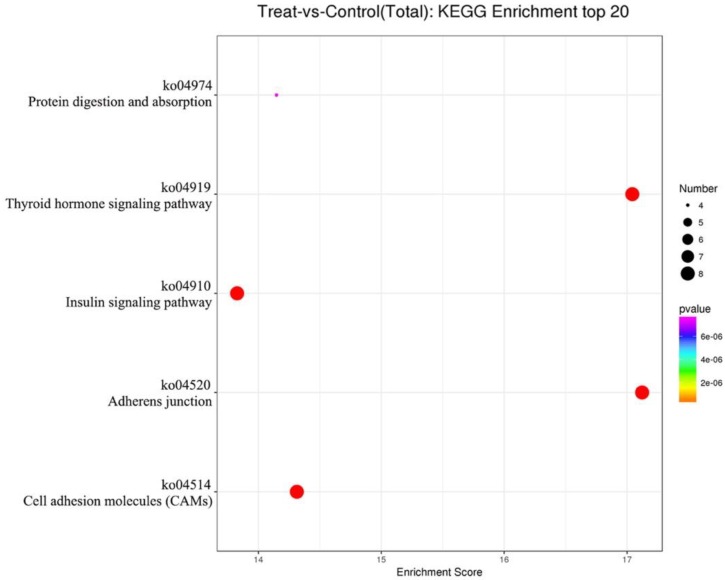
Kyoto Encyclopedia of Genes and Genomes (KEGG) pathway analysis of the target genes of differently expressed known miRNAs.

The target genes of differently expressed known miRNAs in bovine EVs were predicted. The GO enrichments of the potential target genes in three categories (biological process, cellular component and molecular function) are revealed in Figure 6. Biological processes, including histone H acetylation, the regulation of signal transduction by p53 class mediator, T-helper 17 cell lineage commitment, the positive regulation of natural killer cell-mediated cytotoxicity, the positive regulation of interleukin-17 production, the positive regulation of interferon-gamma production, and the regulation of (innate) immune response were potentially affected by the change in diet. The molecular functions of target genes were related to metal ion binding, cell adhesion molecule binding, beta-catenin binding, heparin binding, protein-containing complex binding, chondroitin sulfate proteoglycan binding, DNA binding, DNA-binding transcription factor activity (RNA polymerase II-specific), transcription coregulator activity, and protein tyrosine phosphatase activity. Cytosol and biological membranes, such as nucleoplasm, the plasma membrane, extracellular exosome and the nuclear membrane were the cellular locations of differently expressed miRNAs target genes. The pathway analysis of the target genes of differentially expressed miRNAs was conducted using the KEGG database, and a hypergeometric distribution test was used to calculate the significance of the enrichment of target genes in each pathway entry (Figure 7). Adherens junctions, cell adhesion molecules (CAMs), the thyroid hormone signaling pathway, the insulin signaling pathway, and protein digestion and absorption were the related pathways of the target genes induced by changed EV miRNAs.

## 4. Discussion

The forage-concentrate ratio is traditionally emphasized as a starting point for formulating dairy cattle rations. However, this metric is imprecise when applied to nonforage fiber sources (NFFS), which are high in fiber (like forages) but rapidly passed from the rumen (like concentrates) [13]. NFFS include wheat bran, soybean hull, rice husk, whole cotton seed, and other particular fractions of plants, most often derived from wheat, soybean, rice, corn, oats, or barley [15]. The high-fiber byproducts of the crops are disposable and of little value in most cases. The increase in land use, tighter supplies of food and higher prices of cereal grain result in dramatically higher costs of cattle feeding. In this case, the judicious utilization of NFFS provides a practical strategy for nutritionists to incorporate the feedstuffs into the diets of cattle [13]. It was estimated that the replacement of about 30% of the dry matter grain with soybean hull has no negative effects, either on the fermentation or on the digestion of nutrients, nor on the performance of dairy cows [16]. Ipharraguerre et al. found that the replacement of corn with varying amounts of soyhulls caused a decrease in dry matter intake, but an increase in the milk fat concentration of dairy cattle, with few effects on other production parameters [17]. In this study, we partly replaced alfalfa hay and corn silage with whole cotton seed and soybean hull to reduce the breeding costs and maintain the production of dairy cattle. Besides the favorable price and high availability of soybean hull and whole cotton seed, the latter, as a potentially valuable protein and energy dietary supplement for dairy cows, had high protein, fiber, and oil contents [18]. We found that the reasonable substitution of high-quality feed by high-fiber byproduct feed did not compromise the health and productivity of cattle, whilst sharply reducing feeding costs at the same time (unpublished).

Functioning as fuel and co-factors, micro- and macronutrients, viewed as ’dietary signatures’, can regulate gene and protein expression in the body and, accordingly, influence homeostasis. Emerging genomics tools—such as transcriptomics, proteomics and metabolomics—allow people to identify the regulatory pathways through which these dietary signatures influence homeostasis, being the foundation of nutrigenomics [19]. The outputs of ruminant nutrigenomics studies could help improve the performance of high-yielding dairy cows and modify milk quality for the growing demand for “healthy” food [20]. Toral et al. discussed the specific impact of nutrigenomics on the ruminant physiological processes of milk lipid synthesis [21]. They suggested that a methionine-deficient diet could up-regulate 3% and down-regulate 1% of known miRNAs in mice livers, with miR-182, miR-183, miR-199a-3p, miR-705 and miR-1224 up-regulated, while miR-130a and miR-30c were down-regulated [22]. The ingestion of essential amino acids could increase the expression of miR-499, miR-208b, and miR-23a in human skeletal muscle [23]. High glucose increased the expression of miR-21, which could regulate Akt/TORC1 activity and diabetic kidney disease [24]. N-3 polyunsaturated fatty acids modulated the expression of let-7d, miR-15b, miR-107, miR-191 and miR-324-5p in rat colons [25]. In conclusion, dietary adjustment, including inducing changed intakes of nutrients such as amino acids, fatty acids, carbohydrates, vitamins, and phytochemicals, led to different miRNA expression profiles in various tissues and organs [13]. 

The cardiovascular system is the first formative organ in vertebrate embryo and other organs start to develop adjacent to it, interacting with the preexisting vessels and receiving vascular signals [26]. Therefore, blood is suggested, from the early stage of life development, as the mutual link among different tissues, and lays the foundation of homeostasis. Most, if not all, cell types from different organs can release EVs that then enter into the bio fluids, especially blood [27]. Blood EVs are enriched in miRNAs, with special attributes of their parent cell, or the derived origin, and therefore serve as biomarkers of many diseases [28]. At present, studies on cattle blood EV-miRNAs—which might act as promising early diagnostic biomarkers to improve the health issues in cows, such as mastitis, acidosis, metritis, as well as welfare issues—are very rare [11]. This study showed about 245 miRNAs expressed in bovine serum EVs by RNA sequencing. Similarly, Zhao et al. found that there were about 260 miRNAs in bovine serum EVs using the same method [11]. Moreover, bta-miR-148a, bta-miR-30d, bta-miR-191, bta-miR-423-5p, bta-miR-486, bta-miR-186 and bta-miR-92a were highly expressed in both studies, implying their possible function in the growth and development of cattle. As was predicted in our hypothesis, different diets did change the expression of bovine circulating EV-miRNAs. But the seven differently expressed miRNAs (bta-miR-126-5p, bta-miR-199a-3p, bta-miR-2285bc, bta-miR-2288, bta-miR-3613a, bta-miR-450b, and bta-miR-500) between the two groups were low-expressed on the whole, ranking below 100. To date, there is no information about the target genes of bta-miR-126-5p, bta-miR-2285bc, bta-miR-3613a and bta-miR-450b. The pathway analysis of the target genes of the differentially expressed miRNAs referred to thyroid hormone signaling pathways, insulin signaling pathways, and protein digestion and absorption, indicating that dietary change might lead to different nutrient metabolism and regulate homeostasis.

To date, little was known about the specific destination of the EV-miRNAs in the circulatory system. But we did know most miRNAs were evolutionarily conserved [29]. Therefore, function studies from other species on highly conserved miRNAs might also be applied to cattle. Most highly expressed miRNAs in the serum EVs of this study were immune-related, as reported in bovine or other mammals before. The let-7a-5p and miR-423-5p, highly expressed in this study, were differently expressed in *Mycobacterium bovis* infected cows, suggesting their role in tuning the complex interplay between pathogens and the host immune response [30]. The highly expressed miR-191 in this study was a key regulator of naive, memory, and regulatory T cells, and thereby helped maintain the lymphocyte reservoir, which was necessary to mount productive immune responses [31]. Similarly, miR-30d could control glycosylation by targeting N-acetylgalactosamine transferases, resulting in increased tumor invasion and immune-suppression [32]. The overexpression of miR-125b-5 or miR-99a-5p could promote human γδ T cell apoptosis and inhibit γδ T cell activation, intervening in rapid response to infections as important components of innate immunity [33]. All the findings supported the theory that immune function might be one of the destinations for peripheral blood EV-miRNAs.

## 5. Conclusions

Diets do have some effects on ruminant serum EV-miRNA profiling. Although only several miRNAs were affected in our experiment, for we only changed two feed ingredients, we might safely assume that if the body ingested a completely different diet, there would be more differences. It is of great possibility that people will implement effective dietary strategies to intervene in the physiological state of an animals. It is worth noting that people should get the whole picture of patients and unhealthy animals, especially their dietary status, before taking specific miRNAs as biomarkers. 

## Figures and Tables

**Table 1:** The Total Mixed Ration (TMR) formula of the control group and the tested group.

Items	Experiment Diet	
	Control Group	Tested Group
Ingredient, % of DM		
Alfalfa hay	18.67	9.70
Corn silage	27.39	24.88
Steam-flaked corn	23.20	24.25
Soybean meal	8.22	8.21
Cottonseed meal	9.54	9.54
Beet pulp	5.68	5.68
Distillers dried grains with solubles	3.78	3.77
Whole cotton seed	—	5.72
Soybean hull	—	4.73
Bergagat T300	1.04	1.04
Premix	1.86	1.84
Chemical composition, % of DM		
CP	16.02	16.34
EE	4.20	5.18
RDP(%CP)	58.61	56.64
NDF	31.01	31.84
f NDF	20.92	15.67
peNDF	11.61	11.63
ADF	21.78	22.23
NFC	40.48	38.92
Starch	25.35	25.49
NEL, Mcal/kg	1.61	1.62

**Table 2:** The RNA concentration and miRNAs content (%) of EVs in serum.

Items	Concentration (pg/ μL)	miRNAs Content (%)
Control 1	759.7	76
Control 2	3045.6	75
Control 3	301.1	62
Tested 1	950.8	72
Tested 2	505.0	61
Tested 3	358.8	52

**Table 3:** The number of known miRNAs from *Bos taurus* of all samples.

Item	Control Group			Tested Group		
	Control 1	Control 2	Control 3	Tested 1	Tested 2	Tested 3
Number	230	263	240	258	242	238
Mean	244			246		

**Table 4:** The changes of seven differently expressed known miRNAs in serum EVs.

miRNA-ID	Base Mean	Fold Change	*p*-Value
bta-miR-126-5p	55.80	5.01	0.03
bta-miR-199a-3p	80.07	5.94	0.01
bta-miR-2285bc	17.35	0.01	0.00
bta-miR-2288	17.49	0.02	0.01
bta-miR-3613a	9.23	0.02	0.04
bta-miR-450b	13.45	20.10	0.02
bta-miR-500	69.45	2.52	0.05

Base Mean: The standard read counts of all samples.

## Data Availability

The small RNA sequencing data in this study have been deposited in the NCBI Gene Expression Omnibus [GEO: GSE136806]. (https://www.ncbi.nlm.nih.gov/geo/query/acc.cgi?acc=GSE136806).

## Supplementary Materials

The following are available online at https://www.mdpi.com/2076-2615/9/12/1137/s1, Figure S1, The length distribution of known miRNAs.

## Abbreviations

miRNA	microRNA
EV	Extracellular Vesicle
TEM	Transmission Electron Microscope
GO	Gene Ontology
KEGG	Kyoto Encyclopedia of Genes and Genomes
CAM	Cell Adhesion Molecule
NTA	Nanoparticle Tracking Analysis
TMR	Total Mixed Ration
