# Supplementary figures and images for: Different Diets Change the Expression of Bovine Serum Extracellular Vesicle-miRNAs
Source: Animals (Basel). 2019 Dec 13;9(12):1137. doi: 10.3390/ani9121137 (PMC6940744; doi:10.3390/ani9121137)

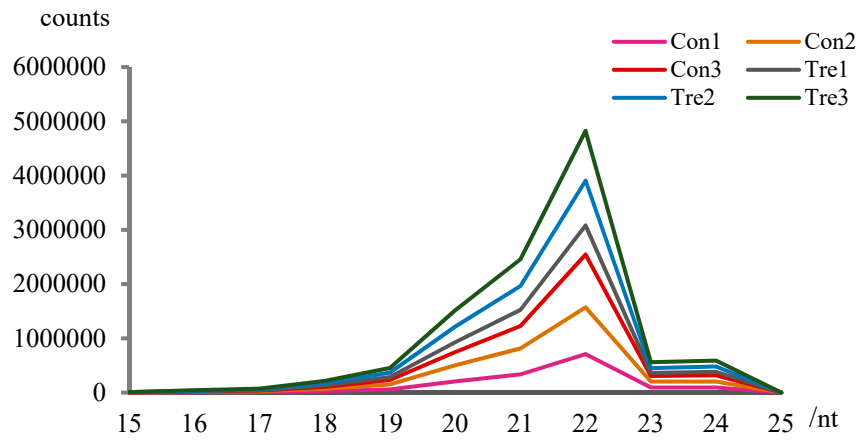

**Supplementary Figure S1.** The length distribution of known miRNAs.

Supplement: Supplementary file 1 [file animals-09-01137-s001.pdf]
